# Misdiagnosed Extranasal Mass: Report of A 2-Year Old Child with Maltreated Rare Nasal Neuroglial Heterotopia

**DOI:** 10.29252/wjps.8.1.122

**Published:** 2019-01

**Authors:** Ezatollah Rezaei, Yavar Shams Hojjati

**Affiliations:** 1 Department of Plastic Surgery, Endoscopic and Minimally Invasive Surgery Research Center, Mashhad University of Medical Sciences, Mashhad, Iran;; 2 Student Research Committee, Mashhad University of Medical Sciences, Mashhad, Iran

**Keywords:** Nasal glioma, Misdiagnosed, Hemangioma, Encephalocele, Outcome

**DEAR EDITOR**

Congenital midline nasal masses, although rare, have some differential diagnoses. Dermoidor epidermoid tumors, encephaloceles, vascular anomalies, and neuroglial heterotopia are the most common diagnoses. Nasal neuroglial heterotopia (formerly known as nasal glioma) is a rare benign congenital lesion which can be at intranasal, extranasal, or mixed anatomic location.^1^^-^^3^ We present a two year old child with a 3×3 centimeters mass over the nasal dorsum. As her parents said, the mass was present from her birth and had constant size and characteristics from then. Previously they had been referred to some physicians and had been diagnosed as hemangioma. 

With such a diagnosis and because of its size and location and probable future visual problems oral corticosteroids had been used, which was ineffective (Figure 1A and B). She was from a normal vaginal delivery without any problem or any positive family history. In systematic examinations, she had no other lesion. It was a partially mobile, incompressible, relatively firm and non-tender mass with normal skin coverage which was located at the nasal dorsum. Nasal airway was patent and there was no intranasal extension. They had a Doppler ultrasonography exam reporting a solid hypoechoic mass with very few vascularity within the mass. In MRI, a CT scan evaluated a well-defined soft tissue mass without any intranasal or intracranial extension. 

**Fig. 1:**
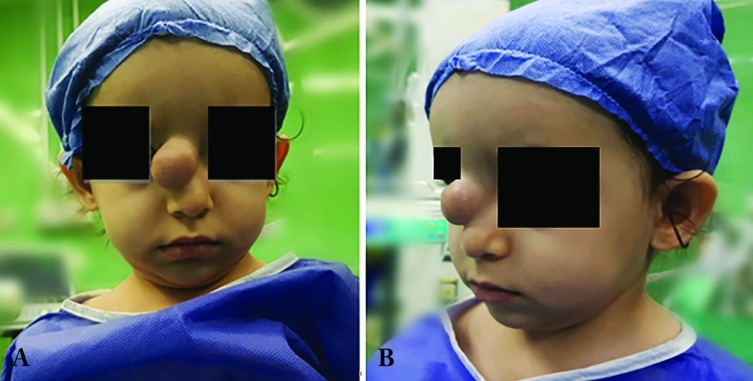
**A: **Frontal view **B:** Lateral view

Small defect in right nasal bone below the mass was also detected (Figure 2A and B). We decided to resect the mass by an extranasal approach. So by an incision over the mass plus resecting the extra skin, we resected the mass which was 25×28 mm and without any obvious capsule. There was a 3×3 mm bone defect underneath the mass and a thin fibrotic band originated from the mass and passing through this defect. Histologic examination showed astrocytic neuroglial cells within fibrous connective tissue and without obvious mitosis (Figure 3A and B).

**Fig. 2:**
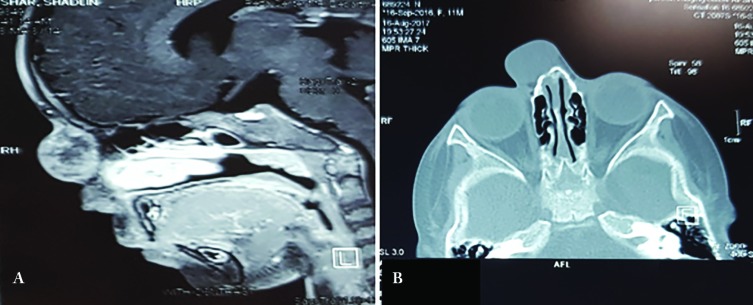
**A:** MRI of the mass. **B:** CT scan of the mass

**Fig. 3:**
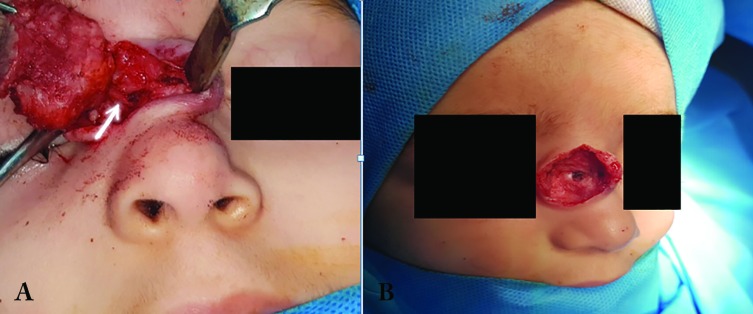
**A:** The fibrotic band passing through the bone defect (white arrow). **B:** Small defect in the right nasal bone

Congenital midline front nasal lesions are very rare benign lesions with an incidence of one in 20000 to 40000 births.^1^ One group of these lesions are nasal glial heterotopias, previously known as nasal glioma. This lesion was first described in 1852^2^ and a total number of about 250 cases had been reported up to 2001.^3^ In 1950 by presenting two case of nasal glioma, Black and Smith defined nasal glioma as a mass composed of glial tissue at or near nasal root which may be connected to brain by a pedicle of same tissue and there was no fluid filled space within the mass.^4^

The exact pathogenesis is not known and there are different theories for development of these lesions. Inappropriate closure of the anterior neuropore, ectopic neural tissue cells, and encephaloceles with lost intracranial connection, are some of these theories.^5^ Nasal glial heterotopia can be seen in different anatomic locations. Sixty percent are extra nasal, 30% are intranasal (nasal cavity, mouth, or pterygopalatine fossa), and 10% are mixed.^6^ A fibrous band connecting them to the intracranial space was seen in 15-20% of cases.^5^ They can cause problems, especially the intranasal mass by its obstructing effect. The extranasal mass except its visibility and aesthetic concerns, are usually asymptomatic, although rare cases of visual problems has been reported.^7^

Preoperative para clinical evaluations are necessary for more reliable diagnosis and better finding the probable lesions’ extensions. Vascular anomalies may be identified by Doppler ultrasonography scan. CT scan and/or MRI should be used, although MRI seems to be the imaging of choice.^8^ Complete excision of the mass is the treatment of choice and inadequate resection may cause recurrence in 4-10% of cases.^9^ Intranasal lesions resection by a transnasal endoscopic approach is the treatment of choice. Extranasal glial heterotopias can be treated by external rhinoplasty approach, or by lateral or medial rhinotomy.^10^ Although congenital nasofrontal masses are rare lesions and nasal glial heterotopias are only a small part of this category, appropriate evaluation can lead to the correct diagnosis and help in choosing the best treatment option. These masses can simply get resected and medical therapies such as systemic corticosteroids should be avoided.

## CONFLICT OF INTEREST

The authors declare no conflict of interest.
